# Practical Points

**Published:** 1894-09-29

**Authors:** 


### PRACTICAL POINTS.

[Questions are invited for this column on any point in the administration of hospitals and asylums which may prove of difficulty or interest to our readers. All communications should be marked "Practical Points," addressed to the Editor of The Hospital, 428, Strand, W.C.]

*The Walls of Hospitals.*—(1) *Is paint or some other colouring best for walls?* (2) *Is there any objection to hanging pictures on the walls?*

*Answer.*—(1) For a new hospital probably the best way of treating the walls of the wards is to paint in oil colour a dado some five or six feet high, finished with a stencilled border or a line, and to distemper a light tint above. When the wards are ready to be redecorated replace the distemper by oil paint and varnish. (2.) The objection to hanging pictures on the ward walls is that they harbour dust. An excellent way of obviating this is to dispense with frames altogether, paste the pictures on to the wall and varnish over the whole. This plan was adopted with great success some years ago by Dr. Percival in the children's ward at the General Infirmary, Northampton.

